# Role of Methylation in Pro- and Anti-Cancer Immunity

**DOI:** 10.3390/cancers13030545

**Published:** 2021-02-01

**Authors:** Ali Mehdi, Shafaat A. Rabbani

**Affiliations:** 1 Department of Human Genetics, McGill University, Montreal, QC H3A 2B4, Canada; ali.mehdi@mail.mcgill.ca; 2 Department of Medicine, Research Institute of the McGill University Health Centre, Montreal, QC H4A 3J1, Canada

**Keywords:** DNA methylation, RNA methylation, S-adenosylmethionine (SAM), cancer, tumor microenvironment, innate immunity, adaptive immunity, T cells, m^6^A

## Abstract

**Simple Summary:**

Epigenetic mechanisms including methylation play an essential role in regulating gene expression not only in cancer cells but also in immune cells. Although role of DNA methylation has been extensively studied in tumor cells in tumor microenvironment (TME), the understanding of transcriptional regulation of pro- and anti-cancer immune cells in TME is beginning to unfold. This review focuses on the role of DNA and RNA methylation in regulating immune responses in innate and adaptive immune cells during their activation, differentiation, and function phase in cancer and in non-cancer pathologies. Uncovering these crucial regulatory mechanisms can trigger discovery of novel therapeutic targets which could enhance immunity against cancer to decrease cancer associated morbidity and mortality.

**Abstract:**

DNA and RNA methylation play a vital role in the transcriptional regulation of various cell types including the differentiation and function of immune cells involved in pro- and anti-cancer immunity. Interactions of tumor and immune cells in the tumor microenvironment (TME) are complex. TME shapes the fate of tumors by modulating the dynamic DNA (and RNA) methylation patterns of these immune cells to alter their differentiation into pro-cancer (e.g., regulatory T cells) or anti-cancer (e.g., CD8+ T cells) cell types. This review considers the role of DNA and RNA methylation in myeloid and lymphoid cells in the activation, differentiation, and function that control the innate and adaptive immune responses in cancer and non-cancer contexts. Understanding the complex transcriptional regulation modulating differentiation and function of immune cells can help identify and validate therapeutic targets aimed at targeting DNA and RNA methylation to reduce cancer-associated morbidity and mortality.

## 1. Introduction

**Figure 1:**
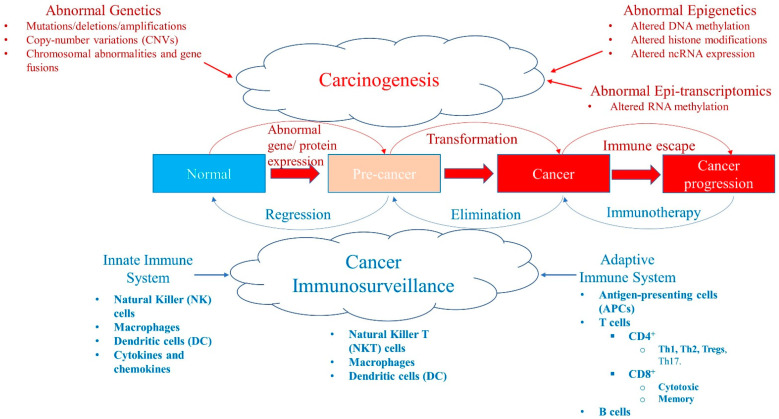
A balance between carcinogenesis and cancer immunosurveillance system. Abnormal genetic modifications such as gene mutations, deletions, amplifications, copy-number variations (CNVs), chromosomal abnormalities, or instability and gene fusions can all result in abnormal expression of genes and proteins leading to transformation of a normal cell into a pre-cancer state and/or cancer stage. Similarly, abnormal epigenetics, such as aberrant DNA methylation patterns, histone modifications, and ncRNA expression (e.g., miRNA) levels, also cause tumorigenesis. Recently, abnormal RNA methylation patterns, such as m^6^A RNA post-transcriptional modifications (epi-transcriptomics), have been shown to result in the initiation and progression of cancer. Although these abnormalities in malignancy promote tumorigenesis, the cancer immunosurveillance system acts as a tumor suppressor working against the formation of pre-malignant and cancer cells. The cancer immunosurveillance system comprises the innate and adaptive immune systems that have various components that help to regress or eliminate tumor cells. However, some immune cells can be pro-tumor, which paradoxically help tumor progression in the tumor microenvironment. Cancer can evolve and escape the immune system by developing immunosuppressive escape mechanisms (such as high expression of PD-L1) that allow it to progress. This state can be reversed with immunotherapy, such as immune checkpoint inhibitors (ICPi).

Epigenetic modifications are heritable changes regulating the cellular gene expression patterns required for the normal development and maintenance of various tissue functions [1,2,3]. Whereas genetic mutations result in the activation/inactivation of certain genes playing a pivotal role in carcinogenesis, abnormalities in the epigenetic landscape can lead to altered gene expression and function, genomic instability, and malignant cellular transformation (Figure 1) [3,4]. The three most studied epigenetic mechanisms that result in cancer are alterations in DNA methylation, histone modification, and non-coding RNA (ncRNA) expression.

### 1.1. DNA Methylation: Writers, Readers, Erasers, and Co-Factors

DNA methylation is the most well-characterized epigenetic mechanism, and was linked to cancer as early as the 1980s [5]. Specific DNA methylation patterns are crucial for parental imprinting, genomic stability, and importantly, regulation of gene expression [6,7]. DNA methylation is the covalent addition of a methyl (-CH3) group at the cytosine (C) base adjacent to 5’ of a guanosine (G) [8,9]. The methyl donor for this methylation reaction is s-adenosylmethionine (SAM). In the human genome, more than 28 million CpG dinucleotides exist, and 60–80% show methylation in any given cell [10]. In contrast, there are specific regions where CpG dinucleotides are enriched, called CpG islands, which are primarily located near gene promoters [10]. Increased methylation at CpG islands is typically associated with gene silencing. However, varying levels of DNA methylation at other regions, including gene bodies, enhancers, 5’ and 3’ UTRs, and partially methylated domains (PMDs), can also differentially affect gene expression to regulate dynamic biological processes [11,12,13,14].

In mammals, the addition of methyl groups to DNA is carried out by “writers”, DNA methyltransferase (DNMT) 1, DNMT3A, and DNMT3B, converting unmodified C into 5-methyl-cytosine (5mC) [15]. DNMT3A and DNMT3B add methyl groups to DNA without template DNA and hence, undertake de novo methylation, whereas DNMT1, maintenance DNMT, adds methyl groups to hemi-methylated DNA by copying DNA methylation patterns from the parental strand to the daughter strand during cell division. DNMTs utilize methyl groups from SAM, which is a universal methyl donor and acts as a co-factor in this reaction [16].

DNA methylation can be recognized by readers including methyl-CpG-binding domain (MBD) proteins, certain transcription factors, and zinc finger (ZNF) proteins [17]. Generally, methylation of the CpG can directly affect gene transcription by interference with the binding of the transcription factors at a regulatory site leading to transcriptional silencing. In addition, DNMTs and MBD proteins can recruit histone modifiers to the methylated promoter region, and stimulate chromatin condensation and gene silencing [15,18,19,20,21].

Methyl groups from DNA can be removed either passively or actively. Active DNA demethylation is performed by “erasers”, called ten-eleven translocation (TET), which remove methyl groups from DNA by oxidizing 5mC into 5hmC (5-hydroxymethyl-cytosine), 5fC (5-formylcytosine), and 5caC (5-carboxylcytosine) [22]. The 5fC and 5caC marks are later identified by thymine DNA glycosylase (TDG), and repaired and replaced by unmodified C. Passive DNA demethylation occurs when DNA methylation maintenance proteins are altered or the DNMT1/UHRF1 complex is unable to read 5hmC, 5fC, or 5caC, leaving C on a newly formed strand unmethylated and, due to multiple rounds of cell division, the original DNA methylation patterns are lost [22].

### 1.2. m^6^A RNA Methylation: Writers, Readers, and Erasers

An emerging crucial layer of post-transcriptional gene regulation, N6-methyladenosine (m^6^A) RNA methylation, plays an essential role in gene expression regulation and development, and human diseases [23,24,25,26,27,28,29,30]. m^6^A is the most common and characterized modification in RNA amongst 150 other post-transcriptional modifications in eukaryotes [23,24,25,26,27,28,29,30]. Alterations in m^6^A RNA methylation and its regulators target different genes in various cancers, including melanoma, acute myeloid leukemia (AML), liver cancer, glioblastoma, and breast and pancreatic cancer (Figure 1) [24,26,27,28,29,30]. m^6^A RNA regulators include writers/methyltransferases, erasers/demethylases, and readers that can add/methylate, remove/demethylate, and read/recognize m^6^A modified sites on RNA, respectively [23,25,26,28]. The major methyltransferases of m^6^A are methyltransferase-like (METTL) 3 and METTL14 complexes that add a methyl group donated from SAM to the 6th Adenosine of the RNAs [23,25,26,28]. In contrast, active demethylation of m^6^A is performed by demethylases AlkB homolog 5 (ALKBH5) or fat mass and obesity-associated (FTO), which remove the methyl groups from the RNA [23,25,26,28]. Readers recognize the m^6^A modification either directly using the YTH domain (e.g., YTH-domain containing reader; YTHDF1/2/3; or YTHDC1/2) or indirectly, which leads to either RNA degradation or enhanced translation of the mRNA [23,25,26].

### 1.3. Immune System: Pro- and Anti-Cancer Immunity

Humans have evolved their immune system, including the innate and adaptive immune systems, to combat a broad range of diseases, including cancer (Figure 1) [31,32,33]. The innate immune system consists of immune cells including natural killer (NK) cells, dendritic cells (DC), macrophages, and neutrophils. The innate immune system is typically the first line of defense, has a nonspecific and immediate response against pathogens, and exhibits germline inheritance [31,32,33]. Innate immune cells use pattern recognition receptors (PRRs), such as toll-like receptors (TLRs), and identify pathogens based on non-specific molecular patterns including single-stranded RNAs or lipopolysaccharide. The adaptive immune system, by comparison, is highly specific and forms the immunological memory. Adaptive immunity comprises lymphocytes, and T and B cells, which produce cytokines and antibodies to counter pathogens [31,32,33]. A large number of extremely diverse but highly specific receptors on T cells—T cell receptors (TCRs)—and B cells—B cell receptors (BCR)—which recognize and differentiate self from non-self antigens are extremely useful in response to foreign pathogens. Long-lasting memory cells generated after pathogen clearance provide a rapid and robust pathogen control upon re-exposure to the same pathogen.

**Figure 2:**
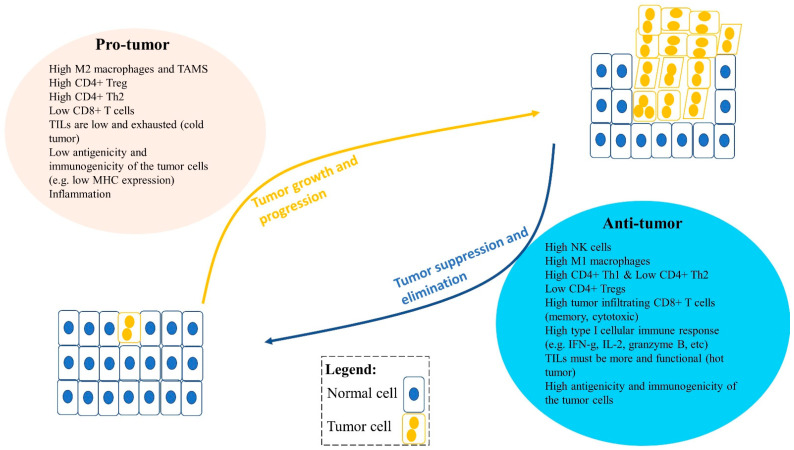
An imbalance between pro-tumor and anti-tumor immune cells and factors in the tumor microenvironment (TME) can lead to tumor growth and progression or tumor suppression and elimination. Pro-tumor immune cells can promote tumor progression, including type II M2 or TAMs (tumor-associated macrophages), regulatory T cells (Tregs), and type II Th2 cells. Moreover, factors that influence tumor progression are low tumor infiltrating lymphocytes (TILs) in the TME, low antigenicity and immunogenicity of tumor cells, and inflammation. Anti-tumor immune cells can reduce tumor growth and suppress tumor progression in the TME. These include CD8+ T cells, type I Th1 cells, NK cells, and type I M1 cells and their type I cytokines such as IFNγ, TNFα, IL-2, and granzyme B. Furthermore, anti-tumor immune factors can also influence tumor suppression, including high infiltration of functional TILs, and greater antigenicity and immunogenicity of the tumor cells, such as high MHC-I expression and tumor-associated antigen expression.

After a century of controversy, it has now been established that a functional cancer immunosurveillance system indeed exists, and acts as a tumor suppressor or killer (Figure 1) [31,32,33,34,35]. Interestingly, both innate and adaptive immune systems can recognize and eliminate malignant cells. Components of the immune system in the tumor microenvironment (TME) can be either anti-tumor, regressing or killing tumor cells; or pro-tumor, helping tumor progression. TME is a complex interaction of tumor cells, immune cells, and stromal cells, and is influenced by various factors including cytokines, chemokines, the extracellular matrix, tissue-specific factors, and inflammation [31,36]. Tumor inhibition or progression depends on TME factors, which can be anti- or pro-tumorigenic. Tumor progression is suppressed or eliminated by the cancer immunosurveillance system; however, tumor cells can evolve and develop mechanisms that allow them to evade or escape the immune system (Figure 1 and Figure 2) [31,36,37]. There are three main immune escape mechanisms: (1) loss of antigenicity—tumor cells increase defects in antigen processing and presentation machinery resulting in lower presentation of antigens to immune cells; (2) loss of immunogenicity—tumor cells produce low levels of immunogenic tumor antigens and high levels of immunosuppressive ligands (e.g., PD-L1); and (3) creating an immunosuppressive TME—tumor cells transform to cause alterations in oncogenes and tumor suppressor genes to increase inflammation and recruitment of pro-tumor immune cells in TME.

Solid tumors typically have immune cells that can be anti-tumor or pro-tumor as a result of factors including differentiation (Figure 2). In summary, pro-tumor factors include high type II M2 macrophages; high CD4^+^ regulatory T cells (Tregs); high type II CD4^+^ Th2 cells; typically low or exhausted tumor infiltrating lymphocytes (TILs) (cold tumor); and low antigenicity and immunogenicity of the tumor cells. In contrast, anti-tumor factors include high NK cells; high type I M1 macrophages; high type I CD4^+^ Th1 cells; low Tregs; high tumor infiltrating CD8^+^ T cells (memory, cytotoxic); high type I cellular immune response (e.g., IFN-g, IL-2, granzyme B); more and functional TILs (hot tumor); and high antigenicity and immunogenicity of the tumor cells (Figure 2) [31,32,33,36,37,38,39,40,41,42,43,44].

Epigenetic mechanisms including miRNAs and histone modifications are crucial for the regulation of the immune system in the TME and has been extensively reviewed [45,46,47,48,49]. DNA methylation also plays an essential role in the differentiation and function of immune cells into various subtypes, and the manner in which these immune cells influence each other in the TME, which ultimately results in tumor progression or suppression. Schuyler et al. [50] carried out analysis of large whole-genome bisulfite sequencing datasets (112 datasets from the BLUEPRINT Epigenome Project) to delineate trends of changes in DNA methylation in different lineages of immune cells, including myeloid and lymphoid cells in TME of various cancer models. Global methylation, in general, increases during macrophage differentiation and activation, whereas it reduces during lymphocyte differentiation (T and B). Numerous studies have also shown methylation changes in the differentiation and activation of pro- or anti-cancer myeloid and lymphoid cells [22,51,52].

The role of methylation in hematopoiesis and in immune disorders is now well established [22,51,52]. The focus of this review is to discuss the role of DNA and RNA methylation (m^6^A) and its regulators in key pro- or anti-cancer immune cells of innate and adaptive immune systems. Examples from other non-cancer immune triggering pathologies are also included. Additionally, the translational potential of targeting methylation with DNA methyltransferase inhibitors (DNMTi), methylating agents such as SAM, and m^6^A RNA demethylase inhibitors in the treatment of liquid and solid cancers is also discussed.

## 2. Role of DNA Methylation in Innate and Adaptive Immunity

### 2.1. Innate Immunity

#### 2.1.1. Dendritic Cells (DCs)

DCs and macrophages are the first innate immunity cell types which are triggered for defense against pathogen invasion. DCs are professional antigen presenting cells (APCs) that are essential for triggering adaptive T cell responses in an antigen-specific manner. DCs can undergo marked changes in their phenotype and function under various stimuli and inflammatory conditions [53]. For instance, DCs can be polarized towards producing specific type of cytokines (e.g., IL-12, IL-23) and Notch ligands (e.g., DLL1/4) to induce different effector CD4 (Th1, Th2, Th17) and CD8 (cytotoxic) T cells [53].

The role of DNA methylation is crucial for regulating differentiation and activation of DCs; however, this has not been fully elucidated, particularly in the TME. Nevertheless, DNA methylation changes have been reported during differentiation of monocytes into DCs and immature DCs (iDCs) into mature DCs (mDCs) [54,55,56,57]. Bullwinkel et al. investigated epigenetic changes occurring at *CD14* and *CD209* gene loci, which are essential for the function of monocytes and DCs, respectively, and found CD14 expression was lost, whereas CD209 expression was elevated, upon differentiation from monocytes to DCs [54]. The reciprocal expression changes in CD14 and CD209 were associated with histone modifications at the *CD14* locus leading to *CD14* silencing, whereas loss of “repressive” histone marks and DNA demethylation at the *CD209* locus resulted in CD209 transcriptional activation. Zhang et al. carried out a comprehensive study of DNA methylation changes at single nucleotide-resolution for human monocytes and monocyte-derived iDCs and mDCs [56]. Several known genes and pathways regulating DC differentiation and maturation were identified. A total of 1608 differentially methylated positions (DMPs) from monocytes to iDCs and 156 DMPs from iDC to mDCs were identified. Major DNA demethylation occurred at the binding sites of the transcription factors of genes involved in DC differentiation and function that ultimately increased transcription of these genes. Moreover, the demethylation was locus-specific, and is associated with changes in DNA methylation regulators, including DNMT1, DNMT3A, DNMT3B, and TET2 [56]. Interestingly, DNA methylation reader, MBD2, in DCs was previously shown to have a dominant role in inducing CD4+ T cells differentiation into the Th2 cell type. Specifically, loss of Mbd2, resulted in reduced phenotypic activation of DCs and capability to initiate Th2 immunity against helminths or allergens [58]. In addition, during IL-4-mediated differentiation from human monocytes to DCs and macrophages, TET2 was identified as the main regulator of DNA demethylation of dendritic cell-specific or macrophage-specific gene sets mostly in intergenic regions and gene bodies [57]. Essentially, the IL-4-JAK3-STAT6 pathway is required for dendritic cell-specific demethylation and expression signature, and STAT6 also prevents demethylation of macrophage-specific genes required for monocyte to macrophage differentiation. Pacis et al. performed a comprehensive epigenome and transcriptome analysis of DCs infected with a live pathogenic bacterium (*Mycobacterium tuberculosis*) [59]. A rapid and active DNA demethylation at distal enhancers was identified that activates master immune transcription factors such as NF-κB and IFN regulatory families [59]. Although the above studies provide strong evidence of DNA methylation regulating monocyte to DC differentiation, and activation of DCs, the role of DNA methylation in the TME needs further characterization.

#### 2.1.2. Macrophages

Macrophages are myeloid cells that have a spectrum of phenotypes in which M1 or M2 subtypes are the extreme ends. M1 cells are “classically activated” by IFNγ, and destroy tumor cells through their production of nitric oxide and type 1 cytokines and chemokines [31,60]. Moreover, M1 act as APCs to activate cytotoxic CD8^+^ T cells in an antigen (Ag)-specific manner. M2 cells are activated by “alternative” pathways via IL-4, IL-13, and/or TGFβ [31,60]. M2 secrete type II chemokines and cytokines, thereby promoting tumor growth and progression. Stromal and tumor-associated factors in the TME can shift macrophages to M2 types, specifically the tumor-associated macrophages (TAMs) type that promotes angiogenesis, tumor progression, and metastasis [60,61,62]. The differentiation from monocyte into macrophages and between the M1 or M2 (or TAMS) phenotype is regulated by DNA methylation at lineage-specific promoter and enhancer regions.

Upon examining global DNA methylation between human monocytes, naïve macrophages, and activated macrophages, Dekkers et al. reported major DNA methylation changes during monocyte to macrophage differentiation [63]. Differential methylation was generally fixed to short regions or single CpGs, and was prevalent at lineage-specific enhancers. The differential methylation was either gain (e.g., *IRF8*, *CEBPB*) or loss (e.g., *PPARG*) of methylation at specific transcription factor binding sites involved in monocyte to macrophage transition. Authors also analyzed different types of activated macrophages and found some genes for lipopolysaccharide (LPS)/IFNγ macrophage-specific activation (e.g., *CCL5*). In another study, the transcriptome and epigenome of human monocytes differentiated into macrophages with colony-stimulating factor 1 (CSF1) identifying several RNAs (mRNA and miRNAs) that are differentially expressed [64]. In addition, 100 differentially methylated regions (DMRs) between monocytes and macrophages were identified in enhancer regions that were uniquely demethylated in macrophages and repressed in monocytes, and were linked to actin cytoskeleton, phagocytosis, and innate immune response [64]. Evidence has shown that both methyltransferases DNMT1 and DNMT3A/B play a vital role in differentiation and macrophage polarization [51]. For instance, knock-down (KD) of DNMT3B in RAW264.7 cells showed a higher polarization towards the M2 macrophage phenotype compared to M1, and leads to suppressed inflammation; the opposite pattern was observed for overexpression of DNMT3B [65]. During chronic inflammation, DNMT1 expression is elevated and has been associated with DNA hypermethylation. A study examined the role of TAMS in DNA methylation of a tumor suppressor gene gelsolin (*GSN*) during gastric cancer progression. Firstly, DNMT1 overexpression was shown to methylate and silence the *GSN* gene, and secondly, DNMT1 overexpression was associated with higher TAMs infiltration in the TME of gastric cancer [66]. Further analysis revealed that TAMs secreted CCL5 that triggered DNMT1 overexpression by activating the JAK2/STAT3 pathway in gastric cells, resulting in GSN silencing and tumorigenesis. In another study, DNMT1 was associated with M1 polarization by silencing the *SOCS1* gene and a subsequent increase in tumor necrosis factor (TNF) and IL-6 production [67]. Furthermore, DNMT1 overexpression was shown to promote M1 activation induced by LPS and IFNγ [67].

In contrast, TET proteins appear to have a role in the downregulation of inflammatory gene expression in normal myeloid cells [22]. In a model of TET2-deficient macrophages and DC, a higher expression of IL-6 was observed upon stimulation [68]. TET2 was shown to reduce IL-6 expression by interacting with Iκbζ (a member of the nuclear IκB family) and binding to the IL-6 promoter region in addition to recruitment of histone deacetylase 2 (HDAC2) [69]. Furthermore, Tet2-deficient mice are more susceptible to septic shock and colitis induced by endotoxin and dextran sulfate sodium (DSS), respectively, both due to elevated IL-6 expression [69]. TET2 expression is elevated in tumor infiltrating myeloid cells of both melanoma patients and mouse models via the IL-1R-MyD88 pathway. Moreover, TET2 acts as an oncogene in melanoma tumorigenesis by suppressing anti-cancer immune cells [70]. This is consistent with the TET protein acting as anti-inflammatory to myeloid cells [22]. Overall, these studies show the role of DNA methylation in regulating monocyte to macrophage differentiation and macrophage polarization.

#### 2.1.3. Natural Killer (NK) Cells

NK cells can directly lyse MHC class I-deficient tumor cells [31,35]. NK cells have activating receptors that identify malignant cells expressing stress-induced ligands (e.g., MICA) [31,35]. NK cells kill the tumor cells by making them undergo apoptosis through either expressing death ligands (e.g., Fas ligand) or by releasing granzymes and perforin [31,35].

The role of DNA methylation in NK cells’ activation or differentiation has not been fully elucidated. However, it was reported that the MHC-I cytotoxicity of NK cells, which is mediated by the KIR (killer cell Ig-like receptor) family, is regulated via methylation. In progenitor cells, KIR genes are silenced via hypermethylation and histone modifications, whereas in KIR-expressing cells, such as NK cells, KIR genes are demethylated and expressed [71]. Furthermore, work with human cytomegalovirus (HCMV) viral infection has shown that, upon infection, subjects have elevated levels of a “memory-like” subtype of NK cells which survive long term and have increased response upon re-exposure of the same pathogen. These memory-like NK cells are characterized by activation of NKG2C, which is in turn epigenetically regulated. In addition, in some HCMV-infected patients, memory-like NK cells were reported to lack B-cell and myeloid signaling proteins such as tyrosine kinase SYK. Further analysis showed that the gene promoter of *SYK* was hypermethylated and SYK expression was downregulated [72]. HCMV-associated NK cells also have low expression of signaling adaptors, including EAT-2, FCER1G, and transcription factor PLZF due to hypermethylation at their DNA [73]. Wiencke et al. examined human naïve vs. activated NK cells’ DNA methylome and found reproducible genome-wide DNA methylation changes [74]. Methylation analysis showed primarily CpG hypomethylation (81% of significant loci) during activation of NK cells. Several previously reported and novel genes or pathways associated with activation of NK cells were identified. The high priority gene *BHLHE40* had high demethylation in activated NK cells, whereas it had low demethylation in naïve NK cells and was shown to be a potential biomarker for NK activation in peripheral blood. Interestingly, increased NK cells and CD8+ T cells tumor infiltration was reported using the DNA methyltransferase inhibitor (DNMTi), AzaC, through type I IFN signaling while reducing the tumor burden of the murine epithelial ovarian cancer model [75]. Histone deacetylase inhibitors (HDACi) lead to further activation of these anti-tumor immune cells and reduction in pro-tumor macrophages in the TME. Furthermore, ligands (such as ULBPs and MICA) of NK cells activating receptor NKG2D, which are essential for NK cell lytic activity, are downregulated in gliomas and hepatocellular carcinoma (HCC) cells via DNA methylation and histone methylation, respectively [76,77]. Indeed, treatment with DNMTi and Enhancer of zeste homolog 2 (EZH2) inhibitor was shown to upregulate NKG2D ligand expression, resulting in the lysis of glioma and HCC cells by NK cells, respectively. These studies show that DNA methylation not only controls the critical gene expression in NK cells that regulates differentiation and activation of NK cells but also genes in cancer cells that regulate NK cell tumor lytic activity.

### 2.2. Adaptive Immunity

Binding of the T cell receptor (TCR) present on T cells to the antigen/MHC complex (signal 1) expressed on APCs is essential for the activation of naive T cells [78]. Additional binding of positive co-stimulatory molecules present on activated APCs, called signal 2 (e.g., CD80/86 and B7RP1 on APCs onto CD28 and ICOS on T cells, respectively), helps in further activation. TCR activation is a multistep process that leads to an intracellular signaling cascade that results in activation, differentiation, and proliferation (clonal expansion) of T cells, and transforms them into effector cells producing cytokines [78]. DNA methylation has a key role in regulating these processes. For instance, upon TCR stimulation of T cells, IL-2 is highly expressed and is required for T cell activation and clonal expansion in mouse [79]. The increase in IL-2 cytokine results from active demethylation at a promoter-enhancer region of the *IL-2* locus upon T cell activation and remains demethylated afterwards [79]. In addition to IL-2 cytokine, DNA methylation also plays an important role in the activation, proliferation, and effector functions of CD4 and CD8 T cells as discussed below.

#### 2.2.1. CD4^+^ T Cells

**Figure 3:**
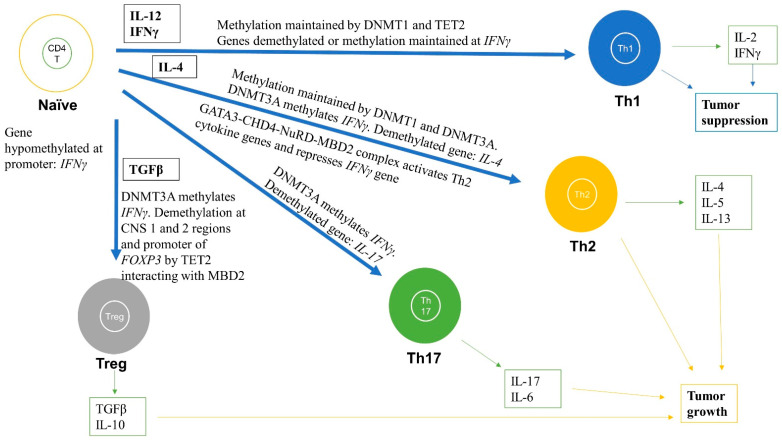
Role of DNA methylation in regulating differentiation and activation of naïve CD4^+^ T cells into effector cells including Th1, Th2, Th17, and Tregs subtypes. DNA methylation changes during differentiation can lead to formation of subtypes of CD4^+^ T cells. The black boxes are cytokines that help in the differentiation and activation process for each subtype. For instance, Th1 are formed when naïve CD4^+^ T cells are stimulated by IL-12 and IFNγ cytokines and the *IFNγ* gene promoter remains hypomethylated and IFNγ is highly expressed. For the Th2 subtype, the *IL-4* gene is demethylated and is highly expressed, whereas *IFNγ* is methylated and repressed. For Th17 cells, the *IL-17* gene is demethylated and highly expressed. For Tregs, *FOXP3* is demethylated at various regions, including promoter and enhancer, thereby markedly increasing FOXP3 expression. These methylation levels are maintained by DNMT1, DNMT3A, and TET2. The green boxes indicate the cytokines released from differentiated cells. These immune cells and released cytokines can further lead to tumor progression or suppression.

CD4^+^ T cells are unique T cells that can, depending on the nature of the Ag signal and type of cytokine stimulation, differentiate into various subtypes including helper T cell 1, 2, and 17 (Th1, Th2, and Th17) and Tregs (Figure 3). Th1 produce type I cytokines, including IL-2 and IFNγ, facilitating optimal expansion, trafficking, and effector functions of CD8^+^ T cells, thereby reducing tumor growth and progression [31,36,37]. In contrast, Th2 produce type II cytokines (IL-4, IL-5, and IL-13) and polarize immunity towards tumor progression [31,36,37]. This differentiation of CD4^+^ T cells into various subtypes is regulated by DNA methylation (Figure 3) [31,36,37]. The differentiated CD4 T cells then regulate downstream immune functions, such as enhancement of CD8 T cells, macrophages, and B cell effector functions, and immunological memory.

Numerous studies have analyzed the methylation status of immune genes and correlated it with immune responses in the TME (Figure 3). Upon antigenic stimulation, naïve CD4^+^ T cells differentiate into Th1 and Th2 by epigenetically activating or silencing a certain set of genes, usually by DNA demethylation and hypermethylation, respectively [80,81,82]. By analyzing the methylation status of a key gene, *IFNG* or *IFNγ*, essential for anti-tumor activity, Janson et al. reported demethylation of the *IFNγ* gene promoter and enhancer, and upregulation of IFNγ in Th1 cells [83]. In contrast, Th2 cells had hypermethylation at the *IFNγ* gene promoter and had low IFNγ expression. Studies show that naïve T cells that develop in the thymus have hypermethylated DNA at enhancer regions of the *IFNγ* and *IL-4* cluster (IL-4, IL-5, IL-13), and methylated H3K27me3 marks [80,81]. These marks limit chromatin accessibility and inhibit transcription of these genes and hence, naïve T cells minimally transcribe these genes. Interestingly, these regions become demethylated in T cell lineages that require expression of these cytokines—for instance, the demethylated promoter of the *IFNγ* gene in Th1 and CD8^+^ T cells [81]. These CpGs are maintained by Dmnt1 as deletion of *Dnmt1* results in global hypomethylation in naïve precursors, including DNA regions which are normally hypermethylated at these cytokine regulatory regions [84]. For instance, in Dnmt1-deficient mice, naïve T cells produce effector cytokines such as IFNγ immediately after activation. This shows that Dmnt1 is required to maintain these hypermethylated regions during T cell development to suppress and induce cytokine gene expression in naïve and active T cells, respectively [84,85]. Indeed, Th1 cells produce 100-times more IFNγ transcripts than naïve T cells but the *IL-4* gene loci are silenced [81].

In contrast, some genes have the opposite pattern, i.e., they have hypomethylation in naïve cells but hypermethylation in differentiated T cells. For example, the *IFNγ* promoter region is unmethylated in naïve CD4^+^ T cells and continues to be hypomethylated upon Th1 cell differentiation; however, upon Th2 cell differentiation, which do not produce IFNγ, the *IFNγ* promoter is methylated via *de novo* DNA methylation by Dnmt3a [86,87]. Moreover, in mouse, *Dnmt3a* deletion in T cells can lead to a complete failure of naïve T cell differentiation into Th2, Th17, and iTreg lineage cells, due to their inability to methylate DNA (de novo) by Dnmt3a at the *Ifnγ* promoter region [88]. Indeed, *Dnmt3a* expression is stimulated upon TCR activation and is recruited to the *Ifnγ* promoter region to carry out methylation in Th2 cells [89]. In addition, deregulated de novo methylation patterns resulted in reduced histone silencing mark (H3K27me3) and increased transcriptionally active histone mark (H3K4me3) upon re-stimulation in the presence of IL-12 [81,88]. Furthermore, Th2 cells produce high amounts of IL-4 as a result of DNA hypomethylation at the *IL4* gene loci and transcriptional activation, whereas in naïve T cells, the *IL4* gene loci are hypermethylated [88]. Finally, during differentiation of naïve CD4^+^ T cells into memory CD4^+^ T cells a global loss of DNA methylation was observed, suggesting a role of DNA methylation in memory CD4^+^ T cell formation [51]. These data suggest that CD4^+^ T cells differentiation into Th1, Th2, Th17, and memory subtypes require DNA methylation changes at gene promoters and enhancers of critical genes such as *IFNγ* and *IL-4* (Figure 3) [36,81,82,83,88].

Strong evidence suggests that the MBD proteins together with the nucleosome remodeling deacetylase (NuRD) complex are essential in regulating DNA methylation-dependent differentiation of T cells [90,91,92]. For instance, loss of either MBD2 or NuRD complex can result in polarization of CD4+ T cells to Th2 cell type. Aoki et al. suggested that the NuRD–MBD2 complex may be required for the demethylation of gene loci encoding cytokines specific for Th2 differentiation [91]. Mechanistically, the chromodomain-helicase-DNA-binding protein 4 (Chd4) subunit of the NuRD–Mbd2 complex forms a complex with Gata3 that both activates Th2 cytokine transcription and represses the Th1 cytokine, IFN-γ, by forming a transcriptional activation complex at Th2 cytokine gene loci and a transcriptional repressive complex at the Tbx21 (encoding T-bet) gene locus in Th2 cells, respectively (Figure 3) [90]. TET proteins have also been linked to the differentiation and function of CD4^+^ T cells (Figure 3). A study analyzing 5-hydroxymethyl-cytosine (5hmC) patterns in CD4^+^ peripheral T cells found a positive correlation between 5hmC alterations at gene bodies of transcription factors, including *Tbx21* and *Gata3*, which drive differentiation into Th1 and Th2 subtypes and their expression levels, respectively [93,94,95]. Similarly, another study suggested similar Th1/2-specific 5hmC alterations during differentiation of human CD4^+^ T cells [93]. In addition, a Tet2 knock-out (KO) mouse model was reported to have Th1 and Th17 cells producing low IFNγ and IL-17, respectively [94]. Overall, these studies suggest that not only DNA methyltransferases (DNMT1 and DNMT3A/B) are required for regulating differentiation of CD4^+^ T cells into various subtypes but also DNA readers and DNA demethylases such as MBD2 and TET proteins, respectively [22,93,94,95].

##### Regulatory T Cells (Tregs)

Tregs can be natural (nTreg), i.e., derived from the thymus, or Ag-induced (iTreg), i.e., differentiated from naïve T cells by TGF-β and IL-2 in the periphery (Figure 3) [31,36,37]. Tregs typically act as pro-tumor, are immunosuppressive, and are associated with poorer prognosis in several cancer types [35,96]. Tregs block the activation of CD8^+^ T cells through expressing cytotoxic T lymphocyte antigen 4 (CTLA4), which is an inhibitory molecule for CD8^+^ T cells [31,96]. In addition, inflammation enhances Treg function because prostaglandin E2 (PGE2) causes differentiation of Tregs. Tregs were also reported to block killing by NK cells, and thus downregulate both adaptive and innate anti-tumor immunity [31,97].

A master regulator switch for Tregs is FOXP3, which is required for its functions (Figure 3). DNA methylation of *FOXP3* together with intergenic CD3G/CD3D regions were utilized as a biomarker for TILs and Treg quantification in several tumor tissues [98]. This DNA methylation-based quantification of immune cells was even comparable to flow cell cytometry and outperformed IHC techniques. Using differential methylation analysis between nTreg, naive CD4^+^ T cells, activated CD4^+^ T cells, and iTreg, Lal et al. found a unique CpG site at the enhancer of *Foxp3* that was unmethylated in nTreg compared to other Tregs that were heavily methylated at this locus [99]. Demethylation by DNMTi (Aza) promoted acetylation of histone 3, and interaction with TIEG1 and Sp1, which ultimately led to upregulation of *Foxp3*. To study Tregs in non-small-cell lung cancer (NSCLC) using a co-culture system, Ke et al. showed demethylation of *FOXP3* in the promoter region increased FOXP3 expression in Tregs, which led to downregulation of immune response in the TME (Figure 3) [100].

Treg-specific demethylated region (TSDR) is a CpG dinucleotide dense region which is within the conserved non-coding sequences 2 (CNS2) located in the first intron of the *FOXP3* gene [101]. DNA demethylation at the TSDR region can discriminate between Tregs and other cell types [102]. Interestingly, using ChIP analysis, Wang et al. showed that MBD2 binds to the TSDR site of the *FOXP3* locus in Tregs [103]. Knocking down Mbd2, in vitro and in vivo, reduced the number of Tregs and impaired Treg-suppressive function (Figure 3). Surprisingly, this was due to increased methylation (>75%) of the TSDR in the Mbd2-/- Tregs because: (i) WT Tregs had a complete TSDR demethylation; and (ii) expressing Mbd2 in Mbd2-/- Tregs rescued the TSDR demethylation. TET proteins are essential for stable Foxp3 expression because they were shown to demethylate the CNS2 region as well as another non-coding sequence, CNS 1, in the *Foxp3* gene (Figure 3) [104,105]. Deletion of Tet2/3 in CD4^+^ T cells of mice led to hypermethylation of CNS1 and 2 in Tregs. Moreover, deletion of *Tet1/2* also resulted in hypermethylation of CNS2 [104,105]. Overexpression of the TET1 catalytic domain in CD4^+^ T cells also resulted in partial demethylation of CNS2 and differentiation of CD4^+^ into iTregs in vitro [106]. TET2 protein may function via interacting with the MBD2 protein because loss of MBD2 resulted in hypermethylation of TSDR in CNS2 [103]. In TME, higher demethylation at the TSDR FOXP3 locus in adjacent normal tissues in colon cancer patient samples were associated with distant metastases and worse recurrence-free survival. The poor survival rates could be due to abnormal recruitment of nTregs in TME [101]. Collectively, these studies show a potential role of DNA methylation in controlling the effector function of Tregs through regulating the expression of the master switch FOXP3 of Tregs.

#### 2.2.2. CD8^+^ T Cells

**Figure 4:**
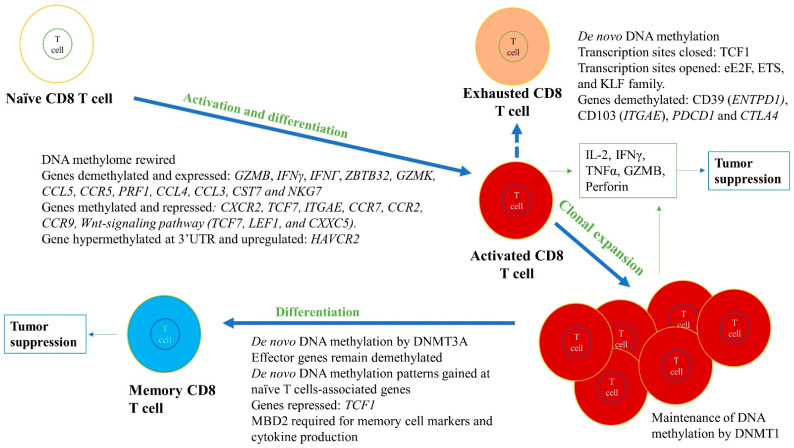
Role of DNA methylation in regulating differentiation and activation of naïve CD8+ T cells into effector cells, including cytotoxic and memory T cell subtypes. DNA methylation changes during differentiation and activation can lead to formation of subtypes of CD8+ T cells. For instance, cytotoxic CD8+ T cells are formed due to whole genome remodeling and expression, and repression of various genes in naïve CD8+ T cells. The genes that are essential for activation, proliferation, and effector functions are demethylated and highly expressed, such as *IL-2*, *IFNG* or *IFNγ*, and *GZMB*, whereas genes that are not required are methylated and repressed (e.g., *TCF7*). Although methylation and gene silencing are usually positively correlated, there are examples of genes that could be methylated and expressed, such as *HAVCR2*, depending upon the precise location of the methylation. In memory CD8+ T cell differentiation, effector genes remain demethylated, whereas methylation at naïve T cell-associated genes are gained and repressed, such as in the case of *TCF1*. These methylation levels are maintained by DNMT1, DNMT3A, and TET2. The green boxes indicate the cytokines released from differentiated cells. These immune cells and released cytokines can further lead to tumor suppression and elimination. However, CD8+ T cells can become exhausted in the TME, highly expressing exhaustive markers such as CD39, CD103, PD-1, and CTLA-4. The exhausted CD8+ T cells are non-functional and produce low amounts of effector cytokines (e.g., IFNγ).

CD8^+^ T cells control tumor growth and kill tumor cells directly in an Ag-specific manner within the TME [31,36,37]. The CD8^+^ T cells, upon recognizing an Ag, can undergo activation and clonal expansion, thereby carrying out effector functions, such as cytokine production (IFNγ, TNFα), and these processes are regulated by DNA methylation (Figure 4) [31,36,37,78].

Epigenetic mechanisms that govern these processes are largely unknown. A study was conducted to delineate these mechanisms and compared Ag-specific naive and effector CD8^+^ T cells after stimulating them with an acute CMV viral infection [107]. The DNA methylome was rewired globally upon effector differentiation of CD8^+^ T cells, and a negative correlation between DNA methylation at gene promoters and gene expression was observed. The DMRs were associated with transcription binding sites and promoters of genes that control effector CD8^+^ T cell function. For instance, DMR at promoters of *Gzmb*, which encodes a serine protease granzyme B essential for cytolytic function, and *Zbtb32*, which encodes a transcription factor induced in activated lymphocytes, was demethylated and had high expression in the effector CD8^+^ T cells compared to naïve cells. In contrast, *Ccr7*, *Ccr2*, *Ccr9*, and *Tcf7*, essential for naïve T cell development and homeostasis, were methylated and had reduced expression. Another study examined *Dnmt3a* KO CD8^+^ T cells and found effector functions to be normal; however, *Dnmt3a* KO T cells developed into fewer terminal effector cells and more memory precursors in a T-cell intrinsic manner. This was due to ineffective repression of *Tcf1* expression by Dnmt3a in *Dnmt3a* KO T cells [108]. The role of Dnmt1 in regulating T cell activation and production of Ag-specific effector and memory CD8^+^ T cells after a viral infection was also investigated. Dnmt1 was knocked-out at the time of activation and *Dnmt1*-/- had marked reduction (>80%) in Ag-specific clonal expansion in effector CD8^+^ T cells but only moderately affected memory CD8^+^ T cells. Even in reduced T cell expansion, the infection was effectively controlled. Thus, Dnmt1 may be required for proliferation of Ag-specific CD8^+^ T cells but not differentiation into effector and memory CD8^+^ T cells [109].

Memory CD8^+^ T cells, which are formed from a subset of effector CD8^+^ T cells after Ag/pathogen clearance, remain in the blood and lymphoid organs for a long time, giving long-term immunity. These memory CD8^+^ T cells also resemble naïve T cells as they have pluripotency and can travel to lymph nodes and the spleen. A study comparing memory CD8^+^ T cells with terminal effector cells found that memory cells formed from effector cells gain de novo DNA methylation patterns at naïve CD8^+^ T cells-associated genes while becoming demethylated at the loci that are effector-specific genes [110]. *Dnmt3a* KO in effector T cells resulted in reduced DNA methylation and quicker re-expression of naïve T cell genes, decreasing the time for memory T cell development. Therefore, in memory CD8^+^ T cells, DNA methylation repression at the naïve-related genes can be reversed and effector genes remain demethylated without the need for memory cells to differentiate, allowing them to become faster effector CD8^+^ T cells upon Ag/pathogen re-exposure.

Long-lived memory CD8^+^ T cells can be identified with a few markers, such as CD127^hi^ and KLRG1^low^. CD127^low^ and KLRG1^hi^ are typically markers for short-lived effector CD8^+^ T cells. Moreover, transcription factors, including T-bet, Eomes, Blimp-1, Bcl-6, Irf4, and Runx3, define the fate of activated CD8^+^ T cells and these are further regulated by DNA methylation. In a mouse model with Tet2-deficient CD8^+^ T cells infected with lymphocytic choriomeningitis virus (LCMV), CD8^+^ T cells differentiated more into long-lived memory cells having gp33-specific memory markers, KLRG1^low^ CD127^hi^, and less into effector short-lived effector cells (CD127^low^ and KLRG1^hi^) [111]. These memory-like cells had markers of central memory cells expressing CD27, CD62L, and CXCR3, and high expression of transcription factor Eomes compared to wild-type Tet2. Furthermore, these memory cells also had superior pathogen control upon re-challenge. Global methylation analysis revealed several DMRs that gained 5mC/5hmC in Tet2-deficient cells versus WT CD8^+^ T cells. These DMRs were present in transcriptional regulator genes known to be vital for effector and memory CD8^+^ T cell differentiation. Pharmacological inhibition of TET2 by 2-HG also showed similar results to genetic *Tet2* KO, such as a decrease in 5hmC and an increase in Eomes and CD62L expression [112]. The role of MBD2 in the differentiation of naïve CD8^+^ T cells into effector and memory cells was determined following LCMV infection. In contrast to Tet2-deficient CD8^+^ T cells, Mbd2-deficient mice had a reduced number of Ag-specific memory CD8^+^ T cells and an effective primary effector CD8^+^ T cell response leading to a rapid viral clearance. Essentially, generation of precursor memory CD8^+^ T cells (IL-7Rα^high^) was delayed and the MBD2 KO memory cells were phenotypically defective with altered memory cell markers (e.g., IL-7Rα, KLRG-1, CD27) and cytokine production, and were unprotective against re-challenge (Figure 4) [113]. These studies suggest a key role of MBD2 and TET proteins in regulating the differentiation of CD8^+^ T cells into memory versus effector cells. Together, the above studies show the crucial role of DNA methylation in differentiation of naïve CD8^+^ T cells into effector cytotoxic CD8^+^ T cells and memory CD8^+^ T cells (Figure 4).

## 3. Role of DNA Methylation in Regulating T Cell Exhaustion

If an Ag is exposed to CD8^+^ T cells for a long time, CD8^+^ T cells can become non-functional or exhausted, resulting in reduced effector functions, such as decreased cytokine production (IFNγ and TNF-α) and/or loss of cytotoxicity (e.g., low granzyme B production). Exhausted T cells generally have high surface expression of multiple inhibitory molecules, such as PD-1, TIM3, LAG3, TIGIT, and 2B4, and transcription factors associated with high PD-1 expression are T-bet, Eomes, and YY1 [114,115,116]. YY1 is a key transcription factor that can regulate the inhibitory molecules PD-1, LAG3, and TIM3 expression, and was shown to have downregulated IL-2 via EZH2 activation, features characteristic of exhausted T cells [114,115,116]. In human patient tumors treated with immune checkpoint inhibitors (ICPi), around 72% of TILs were found to be dysfunctional. These TILs showed different stages of differentiation and interestingly, had higher proliferation rates compared to effector T cells, ruling out the possibility that exhausted T cells have low proliferation rates [114,115,116].

CD8^+^ TILs become exhausted and lose their effector functions in the TME due to numerous factors, such as immunosuppressive mechanisms by tumor cells. Analyzing the transcriptome and methylome of CD8^+^ TILs in the TME of colorectal cancer simultaneously, Yang et al. confirmed tumor-reactive TILs have an exhausted tissue-resident memory signature [117]. They showed tumor-reactive markers CD39 and CD103 of CD8^+^ TILs were demethylated and CD8^+^ TILs had an exhausted phenotype, including high expression of CTLA4, HAVCR2, LAYN, and TOX [117,118]. To delineate changes in methylation from naïve to cytotoxic CD8^+^ T cell phenotype and cytotoxic to exhausted CD8^+^ T cell phenotype, promoter methylation of these cells was compared. Naïve CD8^+^ T cells showed the most promoter demethylation compared to cytotoxic and exhausted T cells; however, essential cytotoxic CD8^+^ T cell effector genes, including *PRF1*, *GZMB*, *IFNG*, *CCL4*, *CCL3*, *CST7*, and *NKG7*, went through hypermethylation to hypomethylation from naïve to cytotoxic CD8^+^ T cell differentiation, respectively [117]. For exhausted T cells, two inhibitory checkpoint markers, *PDCD1* (encoding PD-1) and *CTLA4*, were demethylated within cytotoxic CD8^+^ T cells. Moreover, *LAG3* and *LAYN* were also differentially methylated from naïve to cytotoxic CD8^+^ T cell transition [117]. Therefore, these studies determined that aberrant DNA methylation at certain gene loci could result in T cell exhaustion (Figure 4) [116,117,118].

Interestingly, DNA methylation could determine if T cell exhaustion can be reversed. In chronic LCMV infection, the PD-1 gene promoter of the effector CD8^+^ T cells remained unmethylated, whereas the exhausted T cells showed complete demethylation [116,119]. Furthermore, studies analyzing the chromatin states using transposase-accessible chromatin using sequencing (ATAC-seq) have determined two chromatin states that define exhaustion: one in which T cell factor 1 (TCF1) transcription sites are closed and another in which transcription sites for eE2F, ETS, and KLF family proteins are opened (Figure 4) [120]. Low TCF1 expression is associated with the low effector function of CD8^+^ T cells and nonrenewal of CD8^+^ effector T cells [121]. DNA methylation can, therefore, regulate the state of exhaustion of CD8+ T cells, which, due to the reversable nature of DNA methylation patterns, provides new opportunities for therapeutic intervention.

## 4. Role of m^6^A RNA Methylation in Immunity

m^6^A has various functions, including mRNA stability, translation, splicing, and phase separation, and also takes part in cell differentiation and development [23,24,25,26,27,28,29,30]. These essential functions indicate that m^6^A RNA methylation can potentially regulate immunity. Although the role of m^6^A RNA methylation in immunity has not been fully elucidated, few studies have reported its role in both innate and adaptive immune response [122,123,124,125,126,127,128,129,130,131].

### 4.1. Role of m^6^A RNA Methylation in Innate Immune Response

Certain DNA and RNA molecules can be detected by the innate immune system as non-self entities via PPRs, such as TLRs. For instance, a study investigated the mammalian innate immune response of DCs through stimulation with DNA, RNA, and modified RNAs, including m^6^A-modified RNA [128]. Although DNA containing methylated CpG were not stimulatory, RNA could be stimulatory or not stimulatory depending upon modification on RNA [123,129,130]. Modified RNA, including m^6^A modification exposed to DCs, did not activate their TLR3, TLR7, and TLR8, and led to lower cytokines and activation markers, compared to DC stimulated with unmodified RNA that activated TLRs [123]. Unmodified RNA that is present in bacteria could trigger innate immune response to bacterial infection, whereas highly modified RNA, such as mammalian RNA, would not, indicating a role of RNA modifications in selectively triggering the immune system against pathogens. Indeed, DC are activated via m^6^A RNA modifications and lack of METTL3 can result in lack of DC maturation [123,128,129]. Regulators of m^6^A RNA, METTL14, and ALKBH5 were reported to regulate type I IFN production triggered by dsDNA or HCMV [125,129,130]. Depletion of METTL14 decreased viral replication and induced IFNβ1 mRNA production and stability upon dsDNA and HCMV infection, whereas ALKBH5 depletion had an opposing effect (with the exception of affecting IFNβ1 mRNA stability). This control of IFNβ1 mRNA was due to m^6^A modification at the coding sequence and the 3’ UTR of the *IFNβ1* gene. Another study reported increased interferon-stimulated genes upon METTL3 (m^6^A writer) or YTHDF2 (m^6^A reader) deletion. Specifically, following deletion of METTL3 or YTHDF2, mRNA of IFNβ was modified at m^6^A, increasing its stability [125,129,130]. These studies indicate that m^6^A can play a role in the negative regulation of anti-viral response by dictating increased turnover of IFN mRNAs. One study established a key link of m^6^A to cellular antiviral response by showing that m^6^A induces antiviral immunity as it regulates crucial proteins of innate immunity [131]. Mechanistically, m^6^A demethylase ALKBH5 is recruited by RNA helicase DDX46 to remove m^6^A from 3’ UTRs of genes encoding TRAF3, TRAF6, and MAVS, thereby reducing export of their transcript out of the nucleus and subsequently preventing production of type I IFNs.

### 4.2. Role of m^6^A RNA Methylation in Adaptive Immune Response

m^6^A RNA methylation has also been shown to regulate adaptive immune responses. Similar to DNA methylation regulating differentiation of CD4^+^ T cells into various subtypes, m^6^A RNA methylation was shown to regulate differentiation of CD4^+^ T cells [124]. The authors utilized a conditional KO mouse model (CD4^+^-CRE conditional Mettl3 ^flox^/^flox^) to delete Mettl3 in CD4^+^ T cells [124]. After validating Mettl3 deletion, they checked for thymocyte differentiation or cellularity and found no difference compared to WT mouse. However, the proportion of naïve T cells (CD44^lo^ CD62L^hi^) was higher in spleens and lymph nodes compared to WT. When the function of Mettl3-/- CD4^+^ T cells was compared to WT, they observed normal sensitivity to TCR signaling; however, T helper polarization had abnormalities. For instance, the KO CD4^+^ T cells had a significant reduction in differentiation into Th1 and Th17 cells, but increased differentiation into Th2 cells. In-depth analysis showed that m^6^A targets the mRNA of the IL-7 protein, which regulates T cell homeostatic proliferation and differentiation to various subtypes upon numerous external stimuli. SOCS proteins are adaptors which bind to cytokine receptors, such as the IL-7 receptor, thereby preventing STAT5 and downstream signaling [126,129]. SOCS proteins are produced immediately in response to acute stimuli but are degraded quickly and have short half-lives [126,129]. The m^6^A modification was shown to regulate the degradation of the *Socs* genes, via the IL-7-JAK1/STAT5 signaling pathway, and without m^6^A, Socs mRNA persists, leading to high levels of SOCS proteins and reduced sensitivity to IL-7. This study indicates that m^6^A not only regulates CD4^+^ T cells differentiation but also T cell homeostasis [124]. Using a similar Mettl3 conditional KO mouse model, the authors analyzed the Tregs subset (Mettl3-/- and WT) of CD4^+^ T cells and found that Mettl3 -/- Tregs mice developed severe autoimmune disorders compared to WT, suggesting loss of m^6^A modification can lead to loss of Treg immune suppressive functions [127]. In addition to the writer of m^6^A, readers have shown potential in regulating immune response. As such, compared to WT, a direct reader of m^6^A, Ythdf1 KO mice showed better cross-presentation of tumor antigens in DC and better cross-priming with CD8^+^ T cells, leading to high Ag-specific CD8^+^ T cells in response to tumors [122]. Specifically, binding of Ythdf1 at the m^6^A of transcripts encoding lysosomal proteases lead to increased translation of these lysosomal proteases’ (cathepsins) transcripts in DCs, whereas inhibition of Ythdf1 led to inhibition of these cathepsins, resulting in enhanced cross-presentation by DCs and cross-priming of CD8^+^ T cells by DCs. Indeed, mature DCs were reported to have higher expression of writer complex, including METTL3, than naïve DCs [128]. In addition, patient tumor samples that had low YTHDF1 expression had higher tumor-infiltrating CD8^+^ T cells [122]. Interestingly, mice with Ythdf1 KO showed a better response to ICPi (anti-PD-L1) therapy than the Ythdf1 WT [122].

Collectively, the above studies show the essential role of m^6^A RNA methylation in regulating innate and adaptive immune responses. The role of RNA methylation in immunity is still at its infancy and requires further research for discovery of novel therapeutic targets for its translational potential.

## 5. Targeting Methylation in the Treatment of Human Disease

Alterations in methylation have been strongly associated with the initiation and progression of cancer [132]. Compared to normal control tissues in tumors, DNA hypomethylation occurs at global and gene-specific levels, which results in genomic instability and activation of silenced oncogenes [133]. In contrast, DNA hypermethylation occurs at the promoter regions of tumor suppressor genes (TSGs), which leads to their silencing [133]. With our increasing understanding of the role of methylation in cancer and immunity, further efforts are now aimed at its translational potential to develop new therapeutic strategies that can alter the methylation landscape. Towards these goals, both DNA hypo- and hyper-methylation can serve as viable targets which, unlike genetic changes, are both dynamic and reversible.

### 5.1. Targeting DNA Hypermethylation

Several DNA hypomethylating agents have been developed that target DNA hypermethylation. However, among these DNA methyltransferase inhibitors (DNMTi), 5-azacytidine (Vidaza^®^) and 5-aza-2′deoxycytidine (Decitabine, Dacogen^®^) have been approved by the Food and Drug Administration (FDA) [16]. Because multiple hematologic malignancies are linked to abnormal DNA methylation patterns, DNMTi were first tested in these cancers. Among these, myelodysplastic syndromes (MDS) comprising a group of hematologic disorders derived from abnormal progenitor cells were the first to be evaluated. Patients with MDS have hypoproliferative bone marrow and a risk of developing different forms of acute leukemia [51]. The inhibitor 5-azacytidine was first tested on MDS patients, where it showed improved response rates, lower transformation to acute leukemia, and prolonged survival [134], and 5-aza-2′deoxycytidine showed similar clinical outcomes [135]. Both 5-azacytidine and 5-aza-2′deoxycytidine have also shown success in a clinical setting for acute myeloid leukemia (AML) and chronic myelomonocytic leukemia (CMML) [16].

Following the clinical success of DNMTi with hematologic malignancies, DNMTi were also tested in solid tumors [136,137,138]. Although DNMTi showed a good response in patients with ovarian cancer and non-small cell lung cancer, the response was highly variable and less effective in other solid tumors [136,137,138]. DNMTi has shown the greatest potential in combination with cytotoxic agents or immunotherapies. With cytotoxic agents, DNMTi appear to sensitize tumors and increase the efficacy of conventional cytotoxic agents, even for patients who were previously resistant to the cytotoxic agents alone [139]. Recently, studies have established that malignant cells escape host immune recognition by acquiring an immune evasive phenotype through epigenetically downregulating essential molecules for cancer and immune interactions [35]. For instance, these mechanisms include suppression of tumor associated antigens (TAAs), reducing the expression of many components of antigen processing and presentation machinery (APM), and decreasing co-stimulatory molecules, stress-induced ligands, and death receptors [35]. DNMTi and histone deacetylase inhibitors (HDACi) reverse the immune evasive phenotype, for example, by upregulating the expression of TAAs and APM components on tumor cells, which helps the immune system to recognize and eliminate tumor cells [35,140,141,142]. Additionally, T cell exhaustion can also be reversed using DNMTi in mouse models, resulting in enhanced anti-cancer immunity [143,144]. DNMTi can also trigger a state of “viral mimicry” by activating dsRNAs, thereby increasing type I interferon responses [35,145]. In addition, DNMTi and HDACi increased cytotoxic activity of CD8 T cells and NK cells, and increased these anti-tumor cells’ immune infiltration in the TME while reducing pro-tumor macrophage infiltration in a murine ovarian cancer model [75]. These anti-cancer effects were further elevated in triple combination with ICPi (anti-PD-1), which reduced the tumor burden and provided longest overall survival. Collectively, the above studies indicate priming of the immune system by DNMTi (and HDACi), thereby increasing the efficacy of ICPi therapy.

### 5.2. Targeting DNA Hypomethylation

In cancer, promoter hypermethylation of TSGs and silencing of TSGs resulting in tumorigenesis have been the focus of the last few decades, resulting in the discovery of DNMTi [146,147,148,149]. By comparison, a phenomenon that is relatively underestimated is genome-wide DNA hypomethylation, which occurs in various solid tumors [133,150]. Several studies have also demonstrated that gene-specific and global hypomethylation play a crucial role in the initiation and progression of cancer [7,133]. However, there is still no approved agent that targets DNA hypomethylation. Currently, the most studied approach to target DNA hypomethylation uses SAM. SAM is a natural and universal methyl donor of all methylation reactions [151,152]. As such, SAM donates its methyl group to key cellular components including proteins, nucleic acids (RNA and DNA), lipids, and secondary metabolites to modulate several physiological functions [151,152,153].

Although studies investigating the effect of SAM on the immune system are still lacking, SAM has been shown to modulate the immune system [154,155,156,157,158,159,160,161,162,163,164,165,166,167]. SAM manipulates methylation levels, which further modulates T cell functions by regulating the TCR signaling pathway, impairing Th1/Th2 cytokines release, and decreasing T cell proliferation and activation in autoimmunity [154]. Moreover, SAM reduces IL-1 levels in rats with cecal ligation and puncture. In macrophages, SAM inhibited LPS-induced gene expression via modulation of H3K4 methylation [155]. Similarly, deregulation of SAM levels can result in immune disorders, such as in liver inflammatory diseases. Molecular links between SAM and innate immune functions were reported in which low levels of SAM were shown to affect hepatic PC synthesis and may limit stress-induced protective gene expression upon infection [156]. In addition, SAM prevented upregulation of TLR signaling by blocking the overexpression of TLR2/4 and their downstream partners MyD88 and TRAF-6 in the Mallory–Denk body, forming hepatocytes [157].

Interestingly, studies have shown that SAM is essential for T cell activation and proliferation [154,155,156,157,158,159,160,161,162,163,164,165,166,167]. In activated T cells, both the SAM quantity and the rate of SAM utilization increase dramatically via increased transcription of *MAT2A*, which encodes the catalytic subunit of MATII and is vital for SAM biosynthesis [161,162,164,165]. Blockage of SAM synthesis resulted in blocked T cell proliferation [160]. Furthermore, SAM was shown to be indispensable for T cell proliferation and activation by decreasing both caspase-3 activity and apoptosis in ethanol-related activation-induced cell death (AICD) [159]. Furthermore, SAM was shown to lower the suppressive capacity of Tregs (nTreg cells) by methylating the *FOXP3* gene, thereby reducing its protein and mRNA expression in a dose-dependent manner. SAM was also found to decrease expression of an immunosuppressive cytokine, IL-10, and increase expression of IFNγ [168].

Aberrant methylome is a common consequence of a disrupted SAM cycle associated with transformation of cells towards tumorigenesis [152,169,170]. SAM, which increases DNA methylation, has been shown to cause significant anti-tumor effects in breast, osteosarcoma, prostate, hepatocellular, gastric, colon, and other cancers [151,152,169,170,171,172,173,174]. In addition, SAM levels are depleted by cancer cells through various mechanisms, such as increased conversion of SAM to by-products, which reduces the methylation potential of cancer cells [175,176]. A recent study has shown that an essential immune evasive mechanism used by tumor cells is depriving the CD8+ T cells of SAM and methionine (the pre-cursor of SAM) in the TME. This makes CD8+ T cells non-functional and unresponsive to ICPi [175]. Indeed, we showed that SAM in combination with ICPi (anti-PD-1) significantly reduced tumor volume and weight compared to monotherapy in a syngeneic mouse model of advanced melanoma [177]. This effect was partially due to the elevated activation, proliferation, and cytokine production of CD8 T cells. We also observed increased tumor infiltration of CD8 T cells, a higher number of polyfunctional CD8 T cells, and a lower number of exhausted CD8 T cells in the TME. The above studies show a potential of SAM, a co-factor of methylation, in targeting aberrant DNA methylation patterns in the TME as a novel anti-cancer approach that also enhances anti-cancer immunity. Therefore, the effect of SAM on anti-cancer immunity should be studied comprehensively in future studies.

### 5.3. Targeting m^6^A RNA Methylation

The role of DNA methylation in regulating the immune system and cancer has been the focus of research for more than three decades. Regulation of immunity and cancer by m^6^A RNA methylation is still at its infancy. However, novel studies have shown the potential of targeting RNA methylation in cancer. For instance, FTO inhibition through selective inhibitors, such as Meclofenamic acid (MA), MA2, and R-2-hydroxyglutarate (R-2HG), have shown potent anti-cancer activity in several cancers including AML, glioblastoma multiforme (GBM), and colorectal cancer (CRC) [26,30,178]. In contrast to other RNA demethylase inhibitors, Rhein was identified to be reversibly bound to the FTO catalytic domain via a crystal structure approach and shown to increase m^6^A RNA methylation levels [178,179]. Rhein is attractive as it is a natural compound and selective against FTO and not ALKBH5 [179]. Rhein has shown significant anti-cancer activity in various cancers; however, comprehensive in vivo evidence is still lacking and would require further in-depth studies [180]. Citrate was identified as an ALKBH5 inhibitor via a crystal structure approach; however, the effect of citrate on ALKBH5 demethylase activity in reducing cancer growth and progression is yet to be determined [181].

Although the inhibitors for RNA methylation regulators have been identified, none of them have been tested in a clinical setting. Furthermore, the effect of these pharmacological inhibitors of RNA methylation on the immune system is yet to be determined. Along this line, recently, RNA demethylase FTO was reported to promote tumorigenesis in melanoma and knockdown of FTO-reduced resistance to ICPi (anti-PD-1) therapy [182]. FTO regulates important immune genes (including PD-1, CXCR4, and SOX10 genes) and KD of FTO led to increased mRNA decay of these genes through the m6A reader YTHDF2. Furthermore, KD of FTO sensitized melanoma cells to IFNγ, thereby reducing resistance to anti-PD-1 therapy. Similarly, RNA demethylase ALKBH5 KO showed significant reduction in tumor growth and prolonged mouse survival during ICPi therapy in B16 melanoma and CT26 colon cancer mouse models [183]. This was due to ALKBH5 altering gene expression and splicing that leads to changes in lactate levels in the TME. These metabolic changes result in decreased Treg and MDSCs infiltration in the TME. Interestingly, the authors also tested an ALKBH5 inhibitor and showed similar phenotype to the ALKBH5 KO model. These studies not only show the inhibition of m^6^A demethylases as a potential anti-cancer target but also their potential in anti-cancer immunity within the TME.

## 6. Conclusions

**Figure 5:**
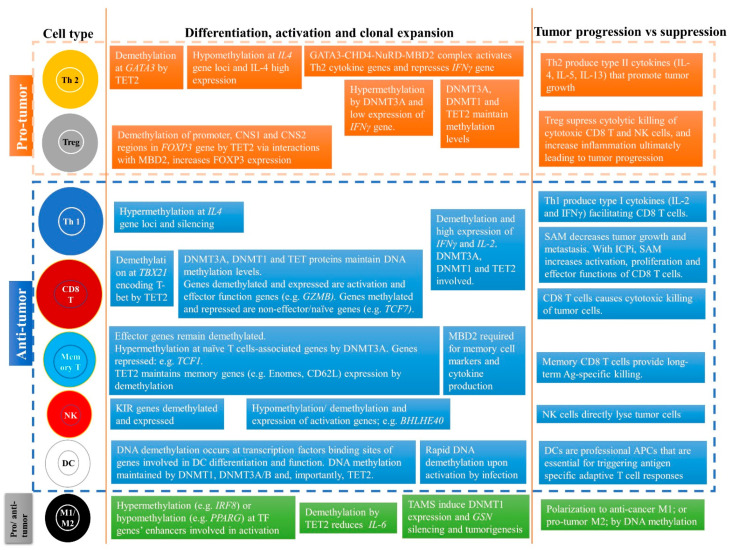
Summary of the role of DNA methylation and co-factor (s-adenosylmethionine, SAM) in regulating differentiation, activation, and proliferation of pro- and anti-cancer immune cells. The pro- or anti-tumor effect of the immune cells in the TME is also described. Abbreviations: Th2, CD4+ helper T cell 2; Tregs, regulatory T cell; Th1, CD4+ helper T cell 1; CD8 T, CD8 cytotoxic T cells; Memory T cells, CD8 memory T cells; NK, natural killer cell; DC, dendritic cell; M1, macrophage M1 subtype; M2, macrophage M2 subtype; TAMS, Tumor associated macrophages; ICPi, Immune checkpoint inhibitors; s-adenosylmethionine, SAM.

The role of DNA and RNA methylation in regulating the differentiation and activity of immune cells within the TME is key to determining the fate of tumor growth or suppression (Figure 5). A pro-cancer TME has immune cells expressing pro-tumor cytokines that lead to tumor growth and progression, whereas the reverse is seen in the anti-cancer TME. Precise methylation patterns change gene expression, leading to specific immune cell subtypes. For instance, DNA demethylation and high expression of *IL4* and *FOXP3* genes occur in Th2 and Tregs, respectively. In contrast, DNA demethylation and high expression of *IFNγ* and *IL2* genes occur in both Th1 and CD8 T cells, which results in a better anti-cancer immune response. Studies should further investigate the effect of DNA and RNA methylation on transcriptional regulation of immune cells along with tumor cells in a time-dependent manner in order to uncover the complexity of the TME at various stages of cancer growth and progression. As explained earlier, the balance between pro- and anti-cancer immune cells within the TME is key to tumor progression or suppression. However, most studies investigating the role of methylation have focused only on one immune cell subtype. Future studies should investigate various immune subtypes simultaneously. These comprehensive studies will provide deeper insights into the interplay between the immune system and cancer, and allow discovery of novel epi-therapies that can enhance the immune system against cancer and other pathologies. Targeting methylation is a particularly attractive anti-cancer strategy because it is dynamic and reversible. For instance, DNMTi that target DNA hypermethylation can also enhance the efficacy of immunotherapies. Similarly, SAM, targeting DNA hypomethylation, has shown profound effects in combination with ICPi. Along the same line, inhibitors of m^6^A RNA demethylases have shown potential in enhancing anti-cancer immunity. However, further comprehensive studies are required to delineate the mechanism of action before these inhibitors can be tested in a clinical setting. In addition, SAM, which donates methyl groups to RNA, has shown significant anti-cancer activity in numerous cancer models by regulating DNA methylation. It is yet to be determined if SAM causes inhibition of tumor growth and metastasis through modulating m^6^A RNA methylation levels. Although the efficacy of epigenetic-based therapeutic strategies targeting tumor and immune cells needs further elucidation, the current state of knowledge provides compelling evidence to suggest that it will be effective in blocking cancer progression and reducing cancer associated morbidity and mortality.

## Data Availability

Not applicable.
